# Determinants of fetal macrosomia among live births in southern Ethiopia: a matched case–control study

**DOI:** 10.1186/s12884-022-04734-8

**Published:** 2022-06-02

**Authors:** Deginesh Dawit Woltamo, Mengistu Meskele, Shimelash Bitew Workie, Abebe Sorsa Badacho

**Affiliations:** 1 Department of Epidemiology and Biostatistics, School of Public Health, College of Health Sciences and Medicine, Wolaita Sodo University, Po.box 138, Sodo, Ethiopia; 2 Department of Reproductive Health and Nutrition, School of Public Health, College of Health Sciences and Medicine, Wolaita Sodo University, Po.box 138, Sodo, Ethiopia; 3 Department of Health Services Management, School of Public Health, College of Health Sciences and Medicine, Wolaita Sodo University, Po.box 138, Sodo, Ethiopia

**Keywords:** Determinants, Fetal macrosomia, Wolaita Sodo town, Southern Ethiopia, Maternal age, Matching

## Abstract

**Background:**

Fetal macrosomia defined as birth weight of 4000 g and above regardless of gestational age and associated with adverse maternal and fetal outcomes, especially among women in developing countries like Ethiopia. Despite the observed burden, there is limited evidence on determinants of fetal macrosomia. This study aimed to identify determinants of fetal macrosomia among live births at Wolaita Sodo town Southern Ethiopia.

**Methods:**

A facility-based matched case–control study design involved 360 singletons deliveries attended at hospitals in Wolaita Sodo town, southern Ethiopia, with 120 cases and 240 controls included. Cases and control were matched by maternal age. Cases were neonates with a birth weight of ≥ 4000, while controls were neonates with a birthweight between 2500gm and less than 4000gm. Data were collected by interviews, measuring, and reviewing mothers' medical documents. Conditional logistic regression analysis was carried to identify the independent predictor variables. Statistical significance was set using a *p*-value < 0.05 and 95% CI for AOR.

**Results:**

Male neonates were four times more likely to be macrosomia than female neonates MAOR = 4.0 [95%CI; 2.25–7.11, *p* < 0.001]. Neonates born at gestational age ≥ 40 weeks were 4.33 times more likely to be macrosomia with MAOR = 4.33 [95%CI; 2.37–7.91, *p* < 0.001]. Neonates born from physically inactive mothers were 7.76 times more likely to be macrosomia with MAOR = 7.76 [95CI; 3.33–18.08, *p* < 0.001]. Neonates born from mothers who consumed fruits and dairy products in their diet frequently were 2 and 4.9 times more likely to be macrosomia MAOR = 2.03 [95%CI; 1.11–3.69, *p* = 0.021] and AOR = 4.91[95%CI; 2.36–10.23, *p* < 0.001] respectively.

**Conclusion:**

Mothers' physical exercise and consumption of fruit and dairy products were significant predictor variables for fetal macrosomia. Hence, health care providers may use these factors as a screening tool for the prediction, early diagnosis, and timely intervention of fetal macrosomia and its complications.

**Supplementary Information:**

The online version contains supplementary material available at 10.1186/s12884-022-04734-8.

## Background

Fetal macrosomia is defined as a total birth weight of 4000 g and above regardless of gestational age or greater than 90 percentile for gestational age [1–5]. The most commonly used threshold of fetal macrosomia in developed countries is the weight above 4500 gm [6]. The grading system used for decision-making regarding operative delivery has suggested grade I 4000—4499 g, grade II 4500 to 4999 g, and grade III for over 5000 g for infants [2, 7].

Globally, macrosomia affects 12% of normal pregnancy and 15%-45% of mothers with gestational diabetes [8]. The magnitude of fetal macrosomia varied from region to region, from one community to another, and has shown temporal changes in the same community due to various factors investigated in different studies [9–14]. Its prevalence is higher in industrialized nations, in affluent countries where their nutritional levels are among women of high socioeconomic status within a given population [6].

In developing countries, fetal macrosomia ranges from 0.5 in India to 15% in Algeria, though there has been a rise in prevalence from 15–25% in the last two decades [10].

Recent studies reveal that the prevalence of fetal macrosomia in Ethiopia is estimated from 6.7% to 19.1% [14–16].

Fetal macrosomia is a significant contributor to obstetric morbidity and mortality. Due to the maternal and neonatal morbidities associated with macrosomia fetuses' pregnancies, such pregnancies are often considered high-risk pregnancies. Macrocosmic baby has a higher threat of developing both short and long term health outcomes in later life; Short term health outcomes: including birth asphyxia, stillbirth, shoulder dystocia, hypoglycemia, skeletal injuries, meconium aspiration, fetal death, and low Apgar score [14, 17, 18]. Similarly, evidence shows that being born macrosomic is associated with health risks in later life, including diabetes mellitus, hypertension, and obesity in adulthood and a higher risk of certain cancers in a future life [8].

Fetal macrosomia is also related to maternal complications like postpartum haemorrhage, prolonged labor, perineal laceration, cesarean delivery, failed instrumental delivery, maternal death, uterine rupture, and wound infection [17–20]The government of Ethiopia has implemented different strategies to improve maternal and newborn health through increasing demand for maternal health services and more accessibility to basic and essential obstetric services, expansion of health facilities, increasing availability of supplies, and deployment of skilled health professionals [21].

Despite the above efforts and strategies, fetal macrosomia is still the significant contributor to maternal and neonatal mortality and morbidity in Ethiopia [15, 16, 22].

Macrosomia can be a more significant obstetric hazard for women in Ethiopia, where undernutrition during childhood can inhibit the growth of the pelvis to its full potential. Pregnancy before the pelvis fully develops joint; delivering a giant baby is distressing to the mother, her baby, obstetrician, and neonatologist. It may lead to unfavourable outcomes during the whole process from pregnancy through delivery and finally after giving birth [13].

Some studies conducted in Ethiopiaidentified gestational age, GDM, sex, weight gain during pregnancy, pre-pregnancy overweight/obesity, maternal age, and parity as predictor variables [12–14].

Since all studies mentioned above were cross-sectional, they were focused on prevalence rate rather than its predictors and focused on clinical factors.

Understanding specific modifiable determinants for macrosomia is crucial for health care providers to prevent macrosomia complications and used to design specific cost-effective interventions. Studies on determinants of macrosomia in Ethiopia is insufficient, and most of those studies were cross-sectional or retrospective focused on clinical factors. Therefore this study was aimed to identify determinants of fetal macrosomia in Ethiopia.

## Methods and materials

### Study area and period

The study was conducted in Wolaita Sodo teaching and referral hospital (WSUTRH) and Sodo Christian general hospital (SCGH), located in Sodo town, Wolaita zone of South Ethiopia. Wolaita Sodo teaching and referral hospital serves about 3 million people. The hospital has one big maternity ward, around 70 beds, about 6000 deliveries per year. Pre-operative and post-operative, inpatient services, abortion care, labour and delivery services, ART services for all pregnant women, and Obstetric/Gynecologic Ultrasound services delivered.

Sodo General Christian hospital is a private hospital in Sodo town, containing four surgical, maternity, medicine/pediatric, and orthopaedic wards. The maternity ward has 25-beds facilities and 750 deliveries per year.

The study was conducted from June to July 2021.

### Study design

The study was a facility-based, matched case–control study. The age of mothers was used for matching, and age strata were created using five-year intervals and a 1:2 case to control ratio.

#### The study population

The cases were macrosomic neonates whose birth weight was ≥ 4000 gm regardless of gestational age and controls: controls were average birth weight neonates whose birth weight was between 2500 and less than 4000gm regardless of gestational age at birth.

## Eligibility criteria

### Inclusion criteria

**For cases:** Neonates with a birth weight of ≥ 4000gm delivered at hospitals of Sodo town during the study period were included in the study as a case.

**For controls:** Selected neonates with birth weights between 2500 and 3999gm, delivered at hospitals in Sodo town during the study period, were included as control after matching for maternal age.

### Exclusion criteria for cases and controls

Those deliveries faced pregnancy complications like abruptio placenta, placenta praevia, multiple pregnancies, and congenital anomalies for both cases, and controls were excluded from the study.

### Sample size determination

The two population proportion formula was used to estimate the sample size required using two different exposure variables, and variables (male sex) with the small odds ratio were selected considering the proportion of exposure among controls 48.8%, and and 8.l% among cases from the study done in Hawassa public health institution, Southern Ethiopia [16]. Based on the following assumption; a ratio of fetal macrosomia cases to controls 1:2, Power 90%, Confidence level 95%, Odds ratio 2.2. total sample size of 366 study participants (122cases and 244 controls) was included in the study adding a 10% non-response rate.

### Sampling procedure and technique

The client registration book of two months before the data collection time was reviewed from two hospitals, and then the total numbers of deliveries during data collection time were estimated which is as (1030 deliveries per two months). The sample size was split between these two hospitals based on the proportionality of their delivery service attendants. A convenient sampling method was used to select cases and controls because pregnant women come to health institutions randomly. Two controls from the source population were selected for every case after matching maternal age until the desired sample size was attained.

### Data collection tools and techniques

We adapted a structured questionnaire from relevant articles and related literature. Data was collected through direct interviews, measurements and supported by reviewing medical records. Age of mothers were used for matching, and age strata was created using five years intervals. For one case, mothers aged between 21–25 years; two control mothers aged 21–25 years were selected, giving a 1:2 case to control ratio. Others were also selected in this way.

The neonate weight was measured within one hour of delivery using a beam balance accurate to 100gm.

The last normal menstrual period (LNMP) was confirmed from her chart and through the interview. Gestational age was estimated based on LNMP and chart review for ultrasound reports.

The dietary habit was assessed based on the number of days per week, based on the Harvard university food frequency questionnaire.

Physical exercise was measured in walking for at least 30 min per day during pregnancy time as a WHO recommendation for pregnant mothers.

History of stillbirth, abortion, and using contraceptive methods used were assessed in terms of the history just before the current pregnancy.

### Operational definitions

Cases: Are neonates whose birth weight were ≥ 4000gm regardless of gestational age.

Controls: Are neonates whose birth weight were between 2500 and 3999gm regardless of gestational age.

Frequently consumption of fruits and dairy products: consumption of fruits and dairy products more than five times per week respectively [23].

Macrosomia is a newborn baby with a birth weight ≥ 4000gm [5].

Average (normal) birth weight: A newborn weighs between 2500 and 3999gm [5].

Birthweight: the fetus or newborn's first weight measured within one hour of birth [5].

Live birth: live birth is the complete expulsion or extraction from its mother of a product of conception, irrespective of the duration of the pregnancy, which, after such separation, breathes or shows any other evidence of life [5].

Physically active pregnant mothers who walk for more than thirty minutes per day [24].

Physically inactive: are those pregnant mothers who walk for less than thirty minutes per day [24].

Birth-interval: is the time interval between live birth and conception of current pregnancy recommended as at least 24 months [25].

Gestational age: is the period between the first day of the last normal menstrual period and date of delivery weeks of pregnancy measured by completed weeks [5]

### Data quality management

Data quality was assured by pretesting on 5% of sample size in Dubo Hospital located in Areka town Wolaita Zone. The data collection tool was prepared in English, translated into local Amharic, and returned to English for consistency. Three data collectors and two supervisors were trained on the content and administration of the questionnaire. The data collectors were three midwife nurses, and two health officers supervised the data collectors. Supervisors checked the filled questionnaire for completeness at the end of each data collection day.

### Data processing and analysis

The collected data were manually checked for completeness and consistency. Then the data was coded and entered into Epidata 4.6.0.2 version and exported to Stata 17 version software for cleaning and further analysis. Data cleaning was performed to check for accuracy, consistency, and mean values. Univariate analysis using frequency technique was performed to describe the data according to the study subjects' essential characteristics. Then the data was expressed in terms of frequency, percentages, and mean. Bivariate conditional logistic regression analysis examined the crude associations between the independent and dependent variables. A variable with a *P*-value of 0.2 and less was taken to multivariable conditional logistic regression to measure the strength of associations and expressed in terms of adjusted odds ratio with 95% confidence interval by adjusting for confounders. Significance was declared at *P*-value ≤ 0.05. Multicollinearity was checked using Variance inflation factor/VIF < 10 running the regress and vif syntaxes in the Stata software. Post estimation command (Hosmer and Lemeshow test) in the logistic regression was run using the estat gof to check the model fitness., The normality of continuous variables was checked using a histogram. Thus, the *p*-value for the Hosmer and Lemeshow chi-square was greater than 0.05, which indicated the model's fitness. The area under the ROC/receiver operating characteristic/ curve was done to classify accuracy.

## Results

### Socio-demographic characteristics of the study population

In this study, a total of 360 participants was interviewed and measured. Overall, 120 cases matched by maternal age with 220 controls taken part in the study to identify risk factors of neonatal macrosomia, producing 98% of response rates. Six (2%) of the participants' interviews were omitted due to incomplete data. This study indicated that 87(72.5) cases and 170(70.8%) participants reside in urban areas. Concerning neonatal sex, 87 (72.5%) cases and 90(37.5%) of controls were males.

Regarding the occupation, the majority of the mothers were housewives, and comparable proportions were reported among cases and controls (56.7% Vs 55.8%).

Concerning educational status, 74(61.7%) of cases and 158(65.8%) controls belongs to secondary and above (Table 1).

**Table 1:** Distribution of Socio-demographic variables vs Neonatal Birth Weight, At hospitals of Wolaita Sodo town, South Ethiopia, 2021

Characteristics	Response	Macrosomia				Crude OR (95% CI)	*P*-value
		Case		Control			
		N	%	N	%		
Sex of new born	Male	87	72.5	90	37.5	4.36(2.70–7.03)	0.001
	Female	150	62.5	33	27.5	1	
Residence	Urban	87	72.5	170	70.8	1.09(0.67–1.78)	0.72
	Rural	33	27.5	70	29.2	1	
Marital status	Married	111	92.5	220	91.7	1.15(0.51–2.59)	0.744
	Others	9	7.5	20	8.3	1	
Religion	Christian	114	95.0	233	97.1	1	
	Muslim	6	5	7	2.9	1.79(0.59–5.47)	0.3
Ethnicity	Wolaita	95	79.2	182	75.8	1.21(071–2.04)	0.489
	Others	25	20.8	58	24.2	1	
Educational status	Primary and below	46	38.3	82	34.2	1.22(0.77–1.94)	0.391
	Secondary and above	74	61.7	158	65.8	1	
Occupation	Housewife	68	56.7	134	55.8	1	
	Others	52	43.3	106	44.2	1.04(0.67–1.62)	0.852
Average monthly family income in Ethiopian Birr	≤ 2620(($ ≤ 87.4)	12	10	28	11.7	1	
	2620–4410($87.4–147.2)	19	15.8	47	19.6	0.92(0.39–2.17)	0.848
	4410–7590($147.2–253)	50	41.7	106	44.2	1.02(0.50–2.28)	0.866
	≥ 7591(≥ $253)	39	32.5	59	24.6	1.53(0.69–3.38)	0.292
Number of family members	< 4 members	49	40.8	117	48.8	1	
	≥ 4 members	71	59.2	123	51.2	1.49(0.91–2.45)	0.111

Obstetric history, medical conditions, and health services utilization of participants.

Eighty-three (69.2%) cases and 130(54.2%) controls were multiparas.

When we look for gestational age, among case 1(0.8%) were born preterm, 101 (84.2%) were born term, and 18 (15.0%) were born post-term. Among controls 4(1.7%) were born preterm, 234 (97.5%) were born term and 2 (0.8%) were born post-term.

Among those who visited ANC utilization services, 75(64.7%) cases and 159(69.4%) controls got dietary counselling.

Among the participants who used contraceptive methods before pregnancy, 61(89.7%) cases and 98(86.7%) of controls used hormonal methods.

Nearly two-thirds of mothers, 234 (65%), were not screened. Among screened mothers, 6(4.8%) had diabetes. Among cases, five (6.8%) were diabetic, and from the controls, one(1.9%) was diabetic (Table 2).

**Table 2:** Distribution of Obstetric, medical, and health services utilization variables vs Neonatal Birth Weight, at hospitals of Wolaita Sodo town, South Ethiopia, 2021

Characteristics	Response	Neonatal macrosomia				Crude OR (95% CI)	*P*-value
		Case		Control			
	N	N	Percent	N	percent		
Parity	Primi Parity	37	30.8	110	45.8	1	
	Multi parity	83	69.2	130	54.2	2.22(1.32–3.74)	0.003
History of abortion before the current pregnancy	Yes	13	10.8	11	4.6	2.56(1.24–5.27)	0.011
	No	107	89.2	229	95.4	1	
History of stillbirth before current pregnancy	Yes	6	5.0	6	2.5	2.15(0.67–6.84)	0.196
	No	114	95.0	234	97.5	1	
Gestational age	Preterm	1	0.8	4	1.7	1	
	Term	101	84.2	234	97.5	1.69(0.19–15.24)	0.641
	Post term	18	15.0	2	0.8	33.86(2.45- 467.7)	0.009
Polyhydramnios during current pregnancy	Yes	7	5.8	11	4.6	1.26(0.48–3.33)	0.64
	No	113	94.2	229	95.4	1	
Hypertension during pregnancy	Yes	12	10.0	23	9.6	1.06(0.51–2.21)	
	No	108	90.0	217	90.4	1	
ANC follow up in current pregnancy	Yes	116	96.7	229	95.4	1.37(0.43–4.37)	0.597
	No	4	3.3	11	4.6	1	
Contraceptive use	Yes	68	56.7	113	47.1	1.55(0.97–2.47)	0.068
	No	52	43.3	127	52.9	1	

### Dietary and lifestyle-related factors

Among the total participants, 102(85%) cases and 200(83.3%) of controls involved in this study frequently consumed cereals.

The proportion of participants who consumed dairy products was higher among cases than controls (29.2% Vs 9.6%).

Regarding consumption of eggs among participants, 21(17.5%) of cases and 27(11.3%) of controls were consumed eggs ≥ five times per week. The proportion of participants who consumed fruits was found to be higher among cases as compared to controls (44.2% Vs 22.1%). When we look for physical exercise during pregnancy, 37(30.8%) of cases and 16(6.7%) of controls were physically inactive (Table 3).

**Table 3:** Distribution of dietary intake and lifestyle-related variables vs. birthweight at hospitals of Sodo town, Wolaita Zone South Ethiopia, 2021

Characteristics	Response	Macrosomia				Crude OR	
		Cases		Control			
		N	Percent	N	percent	95%CI	*P* value
Cereals	Yes	102	85.0	200	83.3	1.17(0.64–2.15)	0.616
	No	18	15.0	40	16.7	1	
Use of roots and tubers	≥ 5 times per day	47	39.2	105	43.8	1	
	< 5 times per day	73	60.8	135	56.3	1.19(0.77–1.87)	0.424
Pulses	≥ 5 times per day	38	31.7	75	31.3	1.04(0.65–1.66)	0.874
	< 5 times per day	82	68.3	165	68.8	1	
dairy	≥ 5 times per day	35	29.2	23	9.6	3.95(2.19–7.12)	0.000
	< 5 times per day	85	70.8	217	90.8	1	
Meat_poultry_fish	≥ 5 times per day	5	4.2	15	6.3	1	
	< 5 times per day	115	95.8	225	93.8	1.53(0.54–4.32)	0.418
nuts_and_seed	≥ 5 times per day	6	5.0	9	3.8	1.33(0.46–3.81)	0596
	< 5 times per day	114	95.0	231	96.3	1	
eggs	≥ 5 times per day	21	17.5	27	11.3	1.69(0.91–3.13)	0.097
	< 5 times per day	99	82.5	213	88.7	1	
Vegetable	≥ 5 times per day	46	38.3	96	40.4	1	
	< 5 times per day	74	61.7	143	59.6	1.11(0.71–1.75)	0.640
fruit	≥ 5 times per day	53	44.2	53	22.1	2.78(1.73–4.45)	0.000
	< 5 times per day	67	55.8	187	77.9	1	
Take additional meal	Yes	89	74.2	164	68.3	1.34(0.82–2.19)	0.243
	No	31	25.8	76	31.7	1	
Exercise time per day during pregnancy	Physically inactive	37	30.8	16	6.7	6.23(3.28–11.83)	0.000
	Physically active	83	69.2	224	93.3	1	

### Predictors of neonatal macrosomia

Bivariate analysis was run in the conditional logistic regression to check the association between dependent and independent variables.

Sex of newborn, history of abortion, history of stillbirth, contraceptive use, physical exercise, family size, average monthly income, parity, gestational age, frequent use of egg, fruit, and dairy products were candidate variables for multiple logistic regression having *p*-value < 0.2 in bivariate analysis.

However, multiple conditional logistic regression analysis showed no difference among cases and controls concerning the history of abortion, history of stillbirth, use of family planning methods, average monthly income, and parity.

Only neonatal sex, physical exercise, gestational age, frequent consumption of fruit and dairy products were independent predictors of neonatal macrosomia at *p* < 0.05.

The sex of neonates has shown a significant association with neonatal birth weight in the study. Male neonates were four times more likely to be macrosomia than female neonates MAOR = 4.0 [95%CI; 2.25–7.11, *p* < 0.001].

Gestational age has shown a significant association with neonatal birth weight in the study. Neonates born at gestational age ≥ 40 weeks were 4.33 times more likely to be macrosomia than neonates from their control groups with MAOR = 4.33 [95%CI; 2.37–7.91, *p* < 0.001].

Physical exercise during pregnancy has shown a significant association with neonatal birth weight in the study. Neonates born from mothers physically inactive mothers (< 30 min per day) during pregnancy were 7.76 times more likely to be macrosomia as compared to neonates born from physically active mothers (≥ 30 min per day)with MAOR = 7.76 [95CI; 3.33–18.08, *p* < 0.001].

Consuming fruits and dairy products have shown a significant association with neonatal birth weight in the study. Neonates born from mothers who consumed fruits and dairy products in their diet frequently (≥ 5 per week) were 2.03 and 4.91 times more likely to be macrosomia as compared to neonates from mothers who consume a fewer amount of fruits and dairy products in their diet with MAOR = 2.03 [95%CI; 1.11–3.69, *p* = 0.021] and MAOR = 4.91 [95%CI; 2.36–10.23, *p* < 0.001] respectively.

In contrast to this, average monthly income, family size, parity, history of abortion before current pregnancy, history of stillbirth before current pregnancy, use of family planning methods, and consumption of eggs were not significantly associated with macrosomia in the final model (Table 4).

**Table 4:** Predictors of neonatal macrosomia among mothers delivered at hospitals in Wolaita Sodo town, 2021

Characteristics	Response	Macrosomia				COR (95%:CI)	AOR (95%:CI	*P*-Value
		Case		Control				
		N	%	N	%			
Sex of new born	Male	87	72.5	90	37.5	4.36(2.70–7.03)	4.0(2.25–7.11)	0.000
	Female	150	62.5	33	27.5	1		
Number of family	< 4 members	49	40.8	117	48.8	1	1	
	≥ 4 members	71	59.2	123	51.2	1.49(0.91–2.45)	1.52(0.50–4.65)	0.464
Average monthly family income in Ethiopian Birr	≤ 2622($ ≤ 87.4)	12	10	28	11.7	1	1	
	2623–4416 ($87.4–147.2)	19	15.8	47	19.6	0.92(0.39–2.17)	0.67(0.21–2.16)	0.507
	4417–7590($147.2–253)	50	41.7	106	44.2	1.02(0.50–2.28)	0.64(0.22–1.82)	0.399
	≥ 7591(≥ $253)	39	32.5	59	24.6	1.53(0.69–3.38)	0.66(0.22–2.02)	0.476
Parity	Primi Parity	37	30.8	110	45.8	1	1	
	Multi parity	83	69.2	130	54.2	2.22(1.32–3.74)	2.33(0.71–7.66)	0.164
Gestational Age	< 40 weeks	28	23.3	131	54.6	1	1	
	≥ 40 weeks	92	76.7	109	45.4	3.85(2.36–6.29)	2.33(2.37–7.91)	0.000
Abortion before the current pregnancy	Yes	13	10.8	11	4.6	2.56(1.24–5.27)	2.56(0.99–6.57)	0.051
	No	107	89.2	229	95.4	1	1	
Stillbirth before current pregnancy	Yes	6	5.0	6	2.5	2.15(0.67–6.84)	1.54(0.38–6.24)	0.548
	No	114	95.0	234	97.5	1	1	
Contraceptive use	Yes	68	56.7	113	47.1	1.55(0.97–2.47)	1.27(0.62- 2.59)	0.518
	No	52	43.3	127	52.9	1	1	
Exercise time per day	< equal30 minutes per day	37	30.8	16	6.7	6.23(3.28–11.83)	7.76(3.33–18.08)	0.000
	> 30 min per day	83	69.2	224	93.3	1	1	
Dairy products	Yes	35	29.2	23	9.6	3.95(2.19–7.12)	4.91(2.36–10.23)	0.000
	No	85	70.8	217	90.8	1		
Fruit	Yes	53	44.2	53	22.1	2.78(1.73–4.45)	2.03(1.11–3.69)	0.021
	No	67	55.8	187	77.9	1		
Eggs	Yes	21	17.5	27	11.3	1.69(0.91–3.13)	0.86(0.39–1.89)	0.718
	No	99	82.5	213	88.7	1	1	

**Fig. 1:**
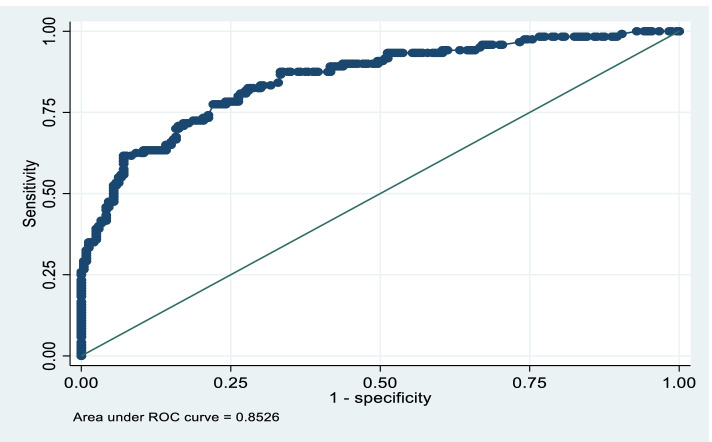
The area under the ROC curve of macrosomia among live births in Sodo town, Southern Ethiopia, 2021

The model fitness was checked by Hosmer–Lemeshow = 4.58 (*p*-value = 0.8017), and 80.56% of variables were correctly classified. The area under the ROC curve was under excellent discrimination (82.56%) Fig. 1.

## Discussion

This study was conducted to assess determinants of macrosomia among neonates delivered at hospitals of Sodo town, Southern Ethiopia, 2021. An institutional-based matched case–control study was employed to answer the research question. The finding of this study revealed that neonatal sex, gestational age, physical exercise, consumption of fruit and dairy products were found to be a positive statistically significant association with macrosomia.

Male neonates were 4.1 times more likely to be macrosomia than female neonates. This result is consistent with reports from cross-sectional studies conducted in Gondar Northern Ethiopia, Hawassa Southern Ethiopia, Cameroon, and retrospective cohort studies in Japan [11, 12, 26, 27]. This might be due to male newborns usually around 150–200 g weights greater than female newborns of the same gestational age near term [28]. Boys were heavier, longer, and had greater head circumference than girls at birth [26, 29]. This might be because of genetic factors and different bodyweight patterns between males and females. However, this study was inconsistent with the study done in Saud found that the proportion of female infants was remarkably higher than males [27]. This might be due to methodological difference, which is descriptive.

Neonates born at gestational age ≥ 40 weeks were 3.7 times more likely to be macrosomia. This was in line with a cross-sectional study in Gondar, Northern Ethiopia, and a case–control study in Tanzania [11, 30]. This might be because an advanced gestational age may cause a large birth weight at delivery by letting growth process in the uterus. Moreover, this is expected as newborns gain weight around 150–200 g near term [29].

Physical exercise was significantly associated with macrosomia. Neonates from physically inactive mothers were 6.8 times more likely to be macrosomia than their control groups. This is consistent with reports from many other studies, case–control studies in Morocco, prospective cohort studies in France and Brazil [23, 31, 32]. This might be due to low-level physical activity during pregnancy may result in gestational weight gain. This, in turn, results in an increased risk of macrosomia.Moreover, it is expected to be a 1 kg increase in the pregnancy weight was associated with a 94 g increase in birth weight [33]. This might be because exercise has been shown to reduce maternal fat storage and fetal adiposity; therefore, it may effectively prevent EGWG and promote healthy birth weight [32]. Physical activity during pregnancy is a modifiable health risk factor and can contribute to the maternal health of women and newborns. In contrast, this study was inconsistent with a study conducted in Canada that indicated that physical exercise during pregnancy was associated with a 2.5 g reduction in an infant's birth weight [34]. This might justify the difference in exercise intensity, which is vigorous exercise.

Neonates born from mothers who consumed fruit frequently were 1.96 times more likely to have been macrosomia than their controls. This aligns with a systematic review conducted in the USA and a prospective study conducted in Japan [35, 36]. This might be because fruits contain many vitamins to promote fetal growth and development [37].

Neonates born from mothers who consumed milk products frequently were 4.1 times more likely to be macrosomia than their controls. This result is consistent with reports from other studies [38, 39]. This might be because milk can promote anabolism and serve as an endocrine signalling system for postnatal growth by activating the nutrient-sensitive kinasemTORC1, thus increasing gestational age and placental and fetal weight [39].

There was no significant difference among parity in this study. This is consistent with reports from other studies on macrosomia [14, 16, 40]. In contrast, there is a significant difference among parity in the prevalence of macrosomia [22, 28, 41, 42]. Although the history of stillbirth [43], miscarriage (45), contraceptive use (45), and hypertension (46) was independently associated with macrosomia in many studies, no such association was found in this study.

### Strength and limitations of the study

#### Strength of the study

- The strength of this study was its study design, a matched case–control and used matched analysis,
- The sample size in this study was large enough, and the findings can be generalized to similar settings in other parts of the country.
- Multiple data collections were used, such as interviews, measurement, and medical record reviews.

#### Limitation of the study

- Any random and systematic measurement error in self-reported data might attenuate the associations observed in this study.
- Self-reported pre-pregnancy body weight, dietary habits, and data regard to menstrual dates may lead to recall bias.
- There is no data on the wealth index; only average monthly income was assessed.

## Conclusion

This study identified multiple predictors of fetal macrosomia. These predictors include; male sex, physical exercise, Gestational age, consumption of fruit and dairy products.

This implies there are modifiable factors such as physical exercise, fruit, and dairy products consumption. Since most of them are modifiable, early recognition and management of these factors at the community level and in ANC providing settings could reduce a significant amount of associated maternal and neonatal complications in this resource limited country.

Health professionals should provide dietary counselling during pregnancy to minimize the consumption of fruit and dairy products, especially after the third trimester, Since the period is vulnerable to birth weight gain.

Health professionals should provide counselling on Physical exercise (e.g., Walking) during the pregnancy period.

Health care providers can use these factors as a screening tool for fetal macrosomia prediction and early diagnosis that allows timely intervention to prevent adverse maternal and neonatal-associated complications.

Gestational diabetic screening and documentation should be taken as standard during ANC visits in these hospitals. 

A large-scale facility-based follow-up study recommended exploring further risk factors associated with fetal macrosomia.

## Supplementary Information

**Additional file 1.** Legend; Supplementary data of all data generated  for neonatal macrosomia among mothers delivered at hospitals in Wolaita Sodo town, 2021. 

## Data Availability

All data generated or analyzed during this study are included in its supplementary information files. Supplementary files has been included in supplementary material section. [Legend; Supplementary data of all data generated for neonatal macrosomia among mothers delivered at hospitals in Wolaita Sodo town, 2021].

## Abbreviations

ACOG: American College of Obstetricians and Gynecologists
ANC: Anti Natal Care
AOR: Adjusted Odds Ratio
BW: Birth weight
BMI: Body Mass Index
EGWG: Estimated Gestational Weight Gain
GDM: Gestational Diabetes Mellitus
GA: Gestational age
LGA: Large for Gestational Age
OR: Odds ratio
WHO: World Health Organization
